# A decision support system (*GesCoN*) for managing fertigation in vegetable crops. Part II—model calibration and validation under different environmental growing conditions on field grown tomato

**DOI:** 10.3389/fpls.2015.00495

**Published:** 2015-07-10

**Authors:** Giulia Conversa, Anna Bonasia, Francesco Di Gioia, Antonio Elia

**Affiliations:** ^1^Department of the Science of Agriculture, Food and Environment, University of FoggiaFoggia, Italy; ^2^Horticultural Science Department, Southwest Florida Research and Education Center, University of FloridaImmokalee, FL, USA

**Keywords:** crop growth modeling, N crop uptake, *Solanum lycopersicum* L., modeling evaluation indices, nitrate vulnerable zone

## Abstract

The *GesCoN* model was evaluated for its capability to simulate growth, nitrogen uptake, and productivity of open field tomato grown under different environmental and cultural conditions. Five datasets collected from experimental trials carried out in Foggia (IT) were used for calibration and 13 datasets collected from trials conducted in Foggia, Perugia (IT), and Florida (USA) were used for validation. The goodness of fitting was performed by comparing the observed and simulated shoot dry weight (SDW) and N crop uptake during crop seasons, total dry weight (TDW), N uptake and fresh yield (TFY). In SDW model calibration, the relative RMSE values fell within the good 10–15% range, percent BIAS (PBIAS) ranged between −11.5 and 7.4%. The Nash-Sutcliffe efficiency (NSE) was very close to the optimal value 1. In the N uptake calibration RRMSE and PBIAS were very low (7%, and −1.78, respectively) and NSE close to 1. The validation of SDW (RRMSE = 16.7%; NSE = 0.96) and N uptake (RRMSE = 16.8%; NSE = 0.96) showed the good accuracy of *GesCoN*. A model under- or overestimation of the SDW and N uptake occurred when higher or a lower N rates and/or a more or less efficient system were used compared to the calibration trial. The in-season adjustment, using the “*SDWcheck*” procedure, greatly improved model simulations both in the calibration and in the validation phases. The TFY prediction was quite good except in Florida, where a large overestimation (+16%) was linked to a different harvest index (0.53) compared to the cultivars used for model calibration and validation in Italian areas. The soil water content at the 10–30 cm depth appears to be well-simulated by the software, and the *GesCoN* proved to be able to adaptively control potential yield and DW accumulation under limited N soil availability scenarios and consequently to modify fertilizer application. The DSSwell simulate SDW accumulation and N uptake of different tomato genotypes grown under Mediterranean and subtropical conditions.

## Introduction

Tomato (*Solanum lycopersicum* L.) is the second most important vegetable crop next to potato in the world. The crop is grown mostly in open field conditions under temperate climates, between the 30^th^ and 40^th^ parallels in both the northern and southern hemisphere. However, with the introduction of modern varieties, tomatoes are increasingly grown in higher temperature, tropical conditions. Tomato production has been reported for about 178 countries. Present production is about 166 million tons fresh fruit produced on 4.7 million hectares. The top 10 leading fruit-producing countries are China, India, Turkey, United States, Egypt, Iran, Italy, Brazil, Spain, and Mexico, accounting for more than three quarters of total world production (FAOSTAT, 2015).

Tomato crops require constant and adequate water and N availability during growth for profitable yield, therefore it benefits from nutrient application through fertigation (Kafkafi and Tarchitzky, 2011). Fertigation allows the uniform application of the right quantity of nutrients to the wetted root volume (Zotarelli et al., 2009), where the active roots are concentrated and this enhances fertilizer use efficiency (Jat et al., 2011). However, as the water soluble nitrate-N move with the wetting front, precise management of irrigation quantity along with rate and timing of N application are critical to achieve the desired results in terms of productivity and nitrogen use efficiency (NUE) while minimizing leaching losses. Tomato crop fertigation mismanagement can lead to (i) insufficient water or N supply to support plant growth, thus resulting in water or N stress for the plant or to (ii) over-irrigation, which may increase N leaching and may negatively affect fruit yield and quality (Kafkafi and Tarchitzky, 2011).

Under specific climate conditions the total amount of nitrogen to be applied during a crop season and the timing depends on the N crops uptake according to crop growth, crop physiological stage, soil type, and N availability in the soil (Hartz and Hochmuth, 1996; Khan et al., 2001).

Computer models that are able to simulate tomato crop growth and N uptake curves under different climatic conditions, soil types and fertility, and crop managements can be very useful tools to increase N and water use efficiency and productivity of the crop. They can help with decision making at the field scale, such as when and how much N and irrigation to apply and to have information on the expected yield.

A simplified decision support systems (DSS) named *GesCoN*, has been developed to account for crop N and water requirements, to manage fertigation at the field scale in open field grown vegetable crops (Elia and Conversa, 2015). It is based on physical sub-models simulating crop dry matter production, crop yield, evapotranspiration, soil moisture, drainage flow, soil nitrogen dynamics, and nitrate leaching. The DSS is an easy-to-use, flexible and adaptive tool, it has specific features and checks for operating in zones designated as nitrate vulnerable zones (NVZs—in accordance with the European Nitrates Directive—1991/676/EEC—and Water Framework Directive—2000/60/EC—objectives), where restrictions in N fertilization application are imposed to prevent the outflow of nitrates from agricultural sources. In these areas, the DSS prevents leaching and adjusts the growth curve in order to comply with the limited N available. *GesCoN* can also be proposed as a Best Management Practice (BMP) tool to guide farmers in the specific irrigation and fertilization recommendations foreseen at various levels in different parts of the world, such as those implemented in each US State in response to the Federal Total Maximum Daily Load mandate described in the Federal Clean Water Act (U.S. Environmental Protection Agency, 2010) or those in the European Eco-Management and Audit Scheme (EMAS).

The current DSS version allows real-time simultaneous (all at once) simulation of many farms having up to 50 sectors each. Each sector may have a different crop, soil, N-fertilizer management, field, and crop management situation. To the best of our knowledge in other software packages to model water and N dynamics in the soil-crop system for managing fertigation in open field conditions, the quantification of crop N demand is only based on tabular data (Moreira Barradas et al., 2012) and, furthermore, no software is available to manage fertigation in NVZs.

The objective of this work was to calibrate the DSS *GesCoN* on open field tomato crop and to validate it under different climate and cultural conditions. With this aim, the DSS *GesCoN* was evaluated by using experimental data collected in trials performed in Italy (under Mediterranean conditions) and in Florida (USA) (under Subtropical conditions), which are two of the most important areas for open field tomato production in the world.

## Materials and methods

### Experimental site and weather data for calibrating and validating *GesCoN*

Data for the model calibration were collected in field trials carried out under Mediterranean climate conditions at Foggia (FG), in Southern Italy, over a period of five consecutive years from 2002 to 2006, hereafter referred to as: FG2002, FG2003, FG2004, FG2005a, and FG2006a, respectively. These data were partially reported in a Ph.D. dissertation (Trotta, 2006) and in published papers (Elia et al., 2006; Rinaldi et al., 2007; Elia and Conversa, 2012).

The model validation was performed using 13 independent datasets collected from experimental trials conducted in three different growing areas. The first area Foggia (FG) was represented by a set of 4 years of experimental data (hereafter referred to as: FG2005b, FG2006b, FG2007, and FG2008) (data from FG2005b, FG2006b partially reported in Conversa et al., 2013) collected on different farms located in the plain of Foggia. For the second area Perugia (PG), a series of 6 years of experimental data was considered (obtained from: Tei et al., 1999, 2002; Benincasa et al., 2006; Onofri et al., 2009) of field trials carried out at Perugia in Central Italy, from 1996 to 1997 and from 1999 to 2002 (hereafter referred to as: PG1996, PG1997, PG1999, PG2000, PG2001, and PG2002, respectively). For the third area Florida (FL), a series of 3 years of data collected in Florida (USA) by Scholberg et al. (2000a,b) was used from field trials carried out at Bradenton in the spring of 1995; Gainesville in the spring of 1996, and Quincy in the fall of 1995 (hereafter referred to as: BRA1995, GAI1996, and QUI1995, respectively). Latitude, altitude, multiannual averaged minimum and maximum air temperatures and soil classification of the FG, PG, and FL sites, as well as other information on the different experimental trials, are reported in Table 1.

**Table 1:** **Main characteristics of the trials from the different sites used for the calibration and the validation of *GesCoN***.

**Trial**	**Latitude**	**Altitude (m a.s.l.)**	**Multiannual averaged temperatures**		**Soil type (USDA classification)**	**Transplanting date (DOY)**	**Harvesting date (DOY)**	**Cultivar name and typology^a^**	**Irrigation System**	**N fertilizer (kg ha^−1^) (application^b^**)	**Plant arrangement^c^**	**Plant density (plants/m^2^)**
			**Min. (°C)**	**Max. (°C)**								
**CALIBRATION TRIALS**												
FG2002	41° 32′ N	75	7.1	25.4	Clay-loam	119	231	Perfectpeel (P)	drip	100 (S2)	TR	2.9
FG2003	41° 32′ N	75	7.1	25.4	Clay-loam	125	233	Perfectpeel (P)	drip	100 (S2)	TR	2.9
FG2004	41° 32′ N	75	7.1	25.4	Clay-loam	139	251	Perfectpeel (P)	drip	100 (S2)	TR	2.9
FG2005a	41° 32′ N	75	7.1	25.4	Clay-loam	161	271	Perfectpeel (P)	drip	200 (F)	TR	2.9
FG2006a	41° 32′ N	75	7.1	25.4	Clay-loam	139	256	Perfectpeel (P)	drip	200 (F)	TR	2.9
**VALIDATION TRIALS**												
FG2005b	41° 32′ N	75	7.1	25.4	Loamy	123	222	Ercole (P)	drip	250 (F)	TR	2.8
FG2006b	41° 32′ N	75	7.1	25.4	Loamy	135	241	Ercole (P)	drip	250 (F)	TR	2.8
FG2007	41° 32′ N	75	7.1	25.4	Silty-clay-loam	137	255	Genius (P)/Talent (P)	drip	300 (F)	TR	3.0
FG2008	41° 32′ N	75	7.1	25.4	Loamy	134	238	PS02313513(P)/Perfectpeel (P)	drip	400 (F)	TR	3.0
PG1996^d^	43°N	450	3.8	22.0	Silt-loam	150	255	PS1296 (P)	drip	200 (S1)	SR	3.2
PG1997^d^	43°N	450	3.8	22.0	Silt-loam	145	254	PS1296 (P)	drip	200 (S1)	SR	3.2
PG1999^d^	43°N	450	3.8	22.0	Silt-loam	139	243	PS1296 (P)	drip	400 (S1)	SR	3.2
PG2000^d^	43°N	450	3.8	22.0	Silt-loam	148	247^f^	PS1296 (P)	drip	200 (S1)	SR	3.2
PG2001^d^	43°N	450	3.8	22.0	Silt-loam	150	241^f^	PS1296 (P)	drip	200 (S1)	SR	3.2
PG2002^d^	43°N	450	3.8	22.0	Silt-loam	154	256^f^	PS1296 (P)	drip	200 (S1)	SR	3.2
BRA1995^e^	27° 29′N	8	15.8	27.8	Sandy	55	143^f^	Sunny (FM)	seepage	200 (S1)	SR	1.1
GAI1996^e^	29° 39′N	37	12.6	27.2	Loamy	74	165^f^	Agriset 761 (FM)	drip	202 (FS)	SR	1.2
QUI1995^e^	30° 35′N	85	10.5	27.0	Loamy-sand	199	283^f^	Solar set (FM)	drip	200 (S1)	SR	1.2

a *P, processing tomato; FM, fresh market tomato*.

b *Modality of N fertilizer application: F, all applied by fertigation throughout the growing season; FS = 40% broadcasted pre-transplanting, 60% by fertigation, S1 = 100% broadcasted before transplanting; S2 = 50% applied before transplanting 50% at the first fruit truss formation*.

c *TR, twin rows; SR, single row*.

d *Reported or calculated from Tei et al. (2002) and Onofri et al. (2009)*.

4 *Reported or calculated from Scholberg et al. (2000a,b)*.

f *As the harvesting date is not reported in the original paper, the value refers to the last sampling date, supposing that it was near to the harvesting date*.

For calibration and validation trials conducted in Foggia, the daily meteorological data files required as input for the simulations, including rainfall, net solar radiation, minimum, and maximum air temperature, wind speed, and maximum and minimum relative humidity, were taken from the weather stations on the experimental farms or in the case of missing data, from the nearest weather station.

For the other trials conducted at Perugia and in Florida, rainfall, minimum, and maximum air temperature daily data files were created by taking data from available net sources which have extensive collections of historical meteorological data (http://www.ilmeteo.it/portale/archivio-meteo for Perugia and ftp://ftp.ncdc.noaa.gov/pub/data/gsod/ for Florida).

### Crop management and N fertilization of experimental trials

Model calibration was performed by selecting the trials having a N fertilization rate ranging from 100 to 200 kg ha^−1^of N, which has been reported to be the range of N supply ensuring the greatest agronomical nitrogen use efficiency (NUE) (fruit DW, for each unit of N applied) for the crop (Scholberg et al., 2000a; Tei et al., 2002; Zotarelli et al., 2009; Elia and Conversa, 2012).

Model validation was performed on trials with N fertilizer rates equal to or higher than 200 kg ha^−1^. When more N rates were available in the same trial, the data relative to the rate closest to 200 kg ha^−1^ was selected.

For the trials conducted in the area of Foggia, five to seven destructive samplings were carried out during the crop cycle to determine the shoot dry weight (SDW) and N uptake (FG2005a and FG2006a). For the Perugia trials the averaged data on SDW accumulation and N uptake (PG1997, PG1999) were kindly provided by the authors. In these trials the SDW data refer to samplings performed at one- to two-week intervals for a total of 10 to 15 samplings depending on the trial. For the trials conducted in Florida the SDW accumulation and N uptake (BRA1995 and QUI1995) were measured with intervals of 1 to 3 weeks, for a total of four to seven samplings depending upon location and associated experimental design. Nitrogen was determined by the Kjeldahl method (Foggia and Perugia) or using Rapid-Flow Analyzer technology (ALPKEM Corp.) (Florida). In FG2005b and FG2006b the gravimetric soil water content was also determined during the crop cycle. Soil was sampled on the row at approximately 20 cm from the center of the plant and at a depth of 10–30 cm, at 2-week intervals. The sampled soil was dried to a constant weight in an oven at 110°C. The weights were multiplied by soil bulk density to calculate the volumetric soil water content (SWC).

The cultivars used were always high yielding hybrid genotypes with determinate habit. Processing tomato cultivars were used in all the Italian trials and fresh market tomato cultivars characterized by large sized fruits in all the Florida ones. In FG2007 and FG2008, where two cultivars were tested, the averaged data were used as the SDW accumulations did not substantially differ between the two cultivars throughout the growing season. In all the simulations it was assumed that cultivars do not differ in phenology phases or the time at which they start flowering, the flowering duration, the time to reach maximum rooting depth, or harvesting maturity, and the same growth function parameters were used regardless of the cultivar or year of study. All trials were conducted in open fields and the crop was established by transplanting. In the Florida trials the growth stage of seedlings was assumed to be younger than that of the Italian trials. The dry weight of seedlings at transplanting was ≈0.2 g/plant in the Florida trials compared 0.5 g/plant in the Italian trials.

Other details on the management of the trials used in the calibration and validation can be found in Table 1 and in the cited papers.

### *GesCoN* calibration and validation

To calibrate and validate *GesCoN* on open field tomato crops 15 parameters were taken or calculated from the literature (L), while 34 were calibrated (C) (Table 2). Four parameters foreseen by the DSS, not being necessary in the specific conditions of the considered tomato trials (*Mulch_Ke*, *Mulch_Kcb*, *Albedo_PT*, and *Alpha_PT*), were not calibrated.

**Table 2:** **Parameters for setting *GesCoN* to work on field grown tomato taken from the literature (L) or calibrated (C)**.

**Parameter**		**Meaning**	**Value**
**Name**	**Type**		
*YldSDW*	L	Dry mass content in the fresh yield at harvest (%)	5.5^a^
*HI*	L	Harvest index	0.66^a^
*RAW*	L	Readily available water (% of *TAW*)	40^b^
*Root_h_max*	L	Maximum root depth of the most efficient part (cm)	40^c^
*Tbase*	L	Base temperature (°C)	10^d^
*Kts*	L	Average dry biomass response factor to water stress	0.49^e^
*E_H_*	L/C	Locally calibrated Hargreaves function coefficient	0.5/0.372^f^
*Albedo_PT*	L	Locally and crop calibrated albedo coefficient	NA
*Alpha_PT*	L	Locally calibrated Priestley–Taylor function coefficient	NA
*a*	L	Critical N curve parameter	4.53^g^
*b*	L	Critical N curve parameter	−0.327^g^
*Kc_ini*	L	Kc value during the initial growth stage of the cycle	0.6^b^
*Kc_mid*	L	Kc value during the middle growth stage	1.15^b^
*Kc_end*	L	Kc value during the late growth stage	0.9^b^
*Kcb_ini*	L	Kcb value during the initial growth stage of the cycle	0.15^b^
*Kcb_mid*	L	Kcb value during the middle growth stage	1.1^b^
*Kcb_end*	L	Kcb value during the late growth stage	0.7^b^
*Root_r_max*	C	Maximum root radius of the most efficient part (cm)	30
*Exp_yld*	C	Expected fresh yield (g/plant)	5300
*Mulch_Ke*	C	*Ke* reduction with film mulching (%)	NA
*Mulch_Kcb*	C	*Kcb* increase with film mulching (%)	NA
*d_SDWstop*	C	Days with low SDW increment before full maturity (no.)	10
*TSMin*	C	Minimum thermal sum for crop maturity (°Cd)^h^	1300
*TSMax*	C	Maximum thermal sum for crop maturity (°Cd)	1660
*Plts_Ref*	C	Reference dry weight of plantlets at transplanting (g)	0.5
*Plts_GR*	C	Initial growth rate (lag phase at plantlets stage) (g d^−1^)	0.015
*β_1_*	C	*β_1_* parameter of the logistic function for shoot growth	12.2874
*β_2_*	C	*β_2_* parameter of the logistic function for shoot growth	6.0894
*β_3_*	C	*β_3_* parameter of the logistic function for shoot growth	−0.0096
*Root_d_max*	C	Number of days to reach maximum values (DAT)^i^	45
*T_M1_*	C	Maximum temperature (°C)	33
*T_M2_*	C	Cut-off temperature (°C)	38
*Flw_beg*	C	Beginning of flowering (°Cd)	250
*Flw_dur*	C	Duration of flowering (°Cd)	250
*Flw_Tmax*	C	Maximum temperature for flowering period (°C)	40
*K_T1_*	C	Shape coefficient for the adjustment of T_*M2*_	3.8112
*K_T_*	C	Shape coefficient for the adjustment of T_*M*2_	3.4909
*K_T_*3	C	Shape coefficient for the adjustment of T_*M*_2	1.7976
*K_SDW1_*	C	Shape coefficient for the adjustment of the expected final SDW	0.9253
*K_SDW2_*	C	Shape coefficient for the adjustment of expected final SDW	0.3733
*N_min_res_1*	C	Minimum N reserve in the soil (kg ha^−1^) in the initial phase	3
*N_min_res_2*	C	Minimum N reserve in the soil (kg ha^−1^) in the mid phase	20
*N_min_res_3*	C	Minimum N reserve in the soil (kg ha^−1^) in the final phase	10
*T_1_*	C	Time in days to complete the initial phase (DAT)	20
*T_2_*	C	Time in days to start the middle phase (DAT)	20
*T_3_*	C	Time in days to complete the middle phase (DAT)	90
*T_4_*	C	Time in days to complete the cycle (DAT)	115
*SC_ini*	C	Soil covered during the initial growth stage of the cycle (%)	10
*SC_mid*	C	Soil covered during the middle growth stage (%)	100
*SC_end*	C	Soil covered during the late growth stage (%)	80
*HP_ini*	C	Average plant height during the initial phase (cm)	20
*HP_mid*	C	Average plant height during the mid-phase (cm)	60
*HP_end*	C	Average plant height during the final phase (cm)	40

a *Calculated based on Elia and Conversa (2012), Tei et al. (2002) and Scholberg et al. (2000a)*.

b *From Allen et al. (1998)*.

c *From Evans et al. (1996) who indicated a root depth of 45 cm for an irrigated tomato crop. This value was further reduced by 5 cm in order to consider the specific conditions of the drip irrigation*.

d *Tbase was set at 10°C for spring-summer cycles, according to Scholberg et al. (2000b), while a value of 6°C was chosen for summer-fall cycles following Calado and Portas (1987), who suggested lower values for areas/climates with higher temperature in the initial stages*.

e *From Patanè et al. (2011)*.

f *In the Florida trials the original E_H_ coefficient was used (0.5), while in the Italian trials the parameter was calibrated on the Foggia area (0.372)*.

g *In the Perugia and Florida trials the values indicated by Tei et al. (2002) *(a = 4.53 and b = −0.327)* were selected, while in the Foggia trials those calibrated by Elia and Conversa (2012) *for Foggia area (a = 3.91 and b = −0.173)*, were used*.

h *DAT, days after transplanting*.

i *°Cd, growing degree day(s)*.

The β_1_, β_2_, and β_3_ growth curve parameters were preliminarily calibrated using the Gauss–Newton method of PROC NLIN in the SAS software, after fitting the logistic function on the observed shoot plant dry weight (SDW).

The parameters used in the functions for the modification of the *T_M2_* threshold value (*K_T1_*, *K_T2_*, and *K_T3_*) and for the redefinition of the expected total dry weight (*K_SDW1_*, *K_SDW2_*), which are the two functions used in the in-season *SDWcheck* procedure for fine tuning the SDW prevision (Elia and Conversa, 2015), were calibrated in two steps. Firstly, the ratio between the predicted and the observed SDW (*CheckPO*) was found for each trial at the DW check time. Then, starting from the day of the check onward, calibrated values were found of *T_M2_* and of the *β_1_*parameter which gave the best fit between the predicted and the observed SDW values (*newT_M2_*, *newβ_1_*). Finally, the parameters *K_T1_*, *K_T2_*, and *K_T3_* of the 3rd order polynomial regression, which relate the change of *newT_M2_* to *CheckPO* values, and the parameters *K_SDW1_*, *K_SDW2_* of the 2nd order polynomial regression which relates the change of *newβ_1_*to *CheckPO* values, were found (Table 2).

The other parameters were calibrated by setting the initial value of the parameters, based on our understanding of crop growth, development and its stress responses, and their subsequent calibration by adjusting them repeatedly after comparing the simulated with the measured results (Table 2).

Validation of the model was focused on the progression of shoot biomass and N uptake with time and on the prediction of the total SDW, total fresh yield and cycle length (time to harvest) as well as on soil water content, which was only tested in two trials.

Depending on the meteorological data available, the ET_0_ was calculated using the FAO Penman–Monteith equation (Foggia) or the Hargreaves-Samani model (Perugia and Florida) (Elia and Conversa, 2015).

As foreseen by *GesCoN*, when running simulations, a “*SDWcheck*” passage (Elia and Conversa, 2015) was performed for each of the SDW experimental data sets used for both calibration and validation. To perform the “*SDWcheck*” the available observed SDW value of the sampling date closest to about one third of the presumable cycle length (from 30 to 50 DAT) was used.

### Data analysis

For both calibration and validation, the performance of the DSS was evaluated, comparing the simulated progression of SDW accumulation and N uptake with the observed data. The statistical evaluation of model accuracy was performed using root mean square error (RMSE), relative root mean square error (RRMSE), RMSE/observation standard deviation ratio (RSR), mean absolute error (MAE), percent bias (PBIAS), and the Nash-Sutcliffe efficiency (NSE). The statistical parameters were defined as follows:

(1) $$\text{RMSE }=\sqrt{\sum_{\text{i }=\;1}^{\text{n}}{\left(S_{i}-O_{i}\right)}^{2}/\text{n}}$$

(2) $$\text{RRMSE}=\text{RMSE}\frac{100}{\bar{O}}$$

(3) $$\text{RSR}=\text{RMSE}/\sqrt{\sum_{\text{i }=\;1}^{\text{n}}{\left(O_{i}-\bar{O}\right)}^{2}}$$

(4) $$\text{NSE}=1-\left[\sum_{1\;=\;1}^{\text{n}}{\left(O_{i}-S_{i}\right)}^{2}/\sum_{\text{i }=\;1}^{\text{n}}{\left(O_{i}-\bar{O}\right)}^{2}\right]$$

(5) $$\text{MAE}=\frac{1}{\text{n}}\sum_{\text{i }=\;1}^{\text{n}}\left(S_{i}-O_{i}\right)$$

(6) $$\text{PBIAS}=\sum_{1\;=\;1}^{\text{n}}\left(O_{i}-S_{i}\right)\text{*100/}\sum_{\text{i }=\;1}^{\text{n}}\left(O_{i}\right)$$

where *S_i_* and *O_i_* are simulated and observed values, respectively, and O is the observed mean value. Both RMSE and MAE describe the difference between model simulations and observations in the units of the variable. Their values close to zero indicate perfect fit, however, values less than half of the standard deviation of the observations may be considered low (Moriasi et al., 2007). RSR standardizes RMSE using the observations standard deviation and varies from the optimal value of 0, which indicates zero RMSE or residual variation and therefore perfect model simulation, to a large positive value. The lower the RSR the lower the RMSE, and the better is the model simulation performance (Singh et al., 2004).

RRMSE provides a measure (%) of the relative difference between simulated and observed data. Simulation results are considered excellent when RRMSE is lower than 10% of the mean, good if between 10 and 20%, fair if between 20 and 30%, and poor if values are greater than 30% of the mean (Jamieson et al., 1991).

Percent bias (PBIAS) measures the average tendency PBIAS, expressed as a percentage, of the simulated data to be larger or smaller than their observed counterparts. The optimal value of PBIAS is 0.0, with low-magnitude values indicating accurate model simulation. Positive values indicate model underestimation bias and negative values indicate model overestimation bias (Gupta et al., 1999).

The Nash-Sutcliffe efficiency (NSE) is a normalized statistic that determines the relative magnitude of the residual variance (“noise”) compared to the measured data variance (“information”). It indicates how well the plot of observed vs. simulated data fits the 1:1 line (Nash and Sutcliffe, 1970). NSE ranges between −∞ and 1.0 (1 inclusive), with NSE = 1, the closer the model NSE efficiency is to 1, the more accurate is the model. Values between 0.0 and 1.0 are generally viewed as acceptable levels of performance, whereas values ≤0.0 indicate unacceptable performance (Moriasi et al., 2007).

The quality of the modeling was also assessed by plotting observed against predicted values. Percent deviation was calculated on total SDW accumulation and N uptake, on fresh yield and on cycle length as:

(7) Deviation=(simulated−observed)/observed×100

To evaluate the response of the model in predicting the crop growth its performance and N uptake, and in adapting the N fertilization schedule, four simulation were performed under different N soil availability scenarios using a tomato crop with the same soil, climatic, and cropping conditions while changing the level of the soil organic matter (SOM): 1.4 or 2.8 g 100 g^−1^ and the condition of being or not being in an NVZ area. For the different N scenarios the predicted crop N uptake and soil N availability during the crop cycle were reported graphically, along with the numerical values of predicted total amount of the N crop uptake, the N fertilizer input, the N mineralization from SOM, the aboveground DW and the fresh yield.

To evaluate the effect of the *SDWcheck* procedure in improving the model accuracy in SDW prediction, each simulation, both in the calibration and in the validation phase, was performed by using or not using the in-season *SDWcheck*. The comparison of the two types of simulations was carried out by dividing the simulations into four groups: (1) FG calibration trials, (2) FG validation trials, (3) PG validation trials, and (4) FL validation trials. For each group, predicted against observed data were plotted and some statistical indices (RRMSE, MAE, PBIAS, and NSE) were calculated to evaluate the effect of the *SDWcheck* procedure in improving the model fitting.

When available, the observed SWCs were graphically compared with those predicted by the software using the same irrigation scheme (timing and water volumes) in the simulations as that followed during the trials.

## Results and discussion

### Calibration

#### Shoot dry weight, yield, and crop cycle length

The calibrated values both for L and C parameters for field grown tomato crop are reported in Table 2.

In the 5 years used for calibrating *GesCoN* for SDW accumulation, the magnitude of the root mean square error (RMSE), representing the differences between observed and simulated values, ranged from 0.60 to 0.81 t ha^−1^(Table 3). The ratio between RMSE and the observations standard deviation (RSR index) which standardizes RMSE, was close to the optimal value zero (0.045/0.079) indicating a very low residual variation and therefore a good model fitting. The RRMSE further confirms the very good model accuracy in predicting SDW accumulation with values falling within the 10 to 15% range, except for the FG2003 trial (18.2%). Moreover, the magnitude of the mean absolute error (MAE) was between 0.48 and 0.70 t ha^−1^ (Table 3) and, as expected, it was lower than RMSE.

**Table 3:** **RMSE, Root mean square error; RRMSE, relative root mean square error, RSR, RMSE-observation standard deviation ratio; MAE, mean absolute error; PBIAS, percent bias; Nash-Sutcliffe efficiency (NSE) for the fittings performed on progression of shoot dry weight (SDW) in calibration and validation trials**.

**Trial**	**RMSE**	**RRMSE**	**RSR**	**MAE**	**PBIAS**	**NSE**
**CALIBRATION**						
FG2002	0.803	14.84	0.060	0.577	−4.41	0.975
FG2003	0.818	18.15	0.079	0.704	−11.50	0.968
FG2004	0.671	13.89	0.066	0.537	−1.91	0.979
FG2005a	0.601	9.54	0.045	0.481	1.30	0.986
FG2006a	0.715	11.81	0.055	0.478	7.35	0.979
**VALIDATION**						
FG2005b	1.256	25.84	0.094	0.809	0.99	0.939
FG2006b	1.499	20.12	0.087	1.076	13.92	0.979
FG2007	1.272	13.96	0.129	1.171	12.85	0.916
FG2008	1.044	14.71	0.084	0.849	10.59	0.968
PG1996	0.812	16.20	0.076	0.624	−11.93	0.948
PG1997	0.658	11.23	0.045	0.478	−7.07	0.977
PG1999	0.615	10.05	0.039	0.470	1.13	0.983
PG2000	1.029	14.93	0.058	0.882	−0.61	0.956
PG2001	0.742	16.23	0.065	0.571	−12.47	0.964
PG2002	0.985	16.50	0.076	0.611	9.43	0.936
BRA1995	0.500	20.67	0.070	0.357	7.36	0.966
GAI1996	0.392	15.16	0.072	0.216	8.36	0.974
QUI1995	0.436	16.03	0.096	0.316	11.59	0.982

**Figure 1:**
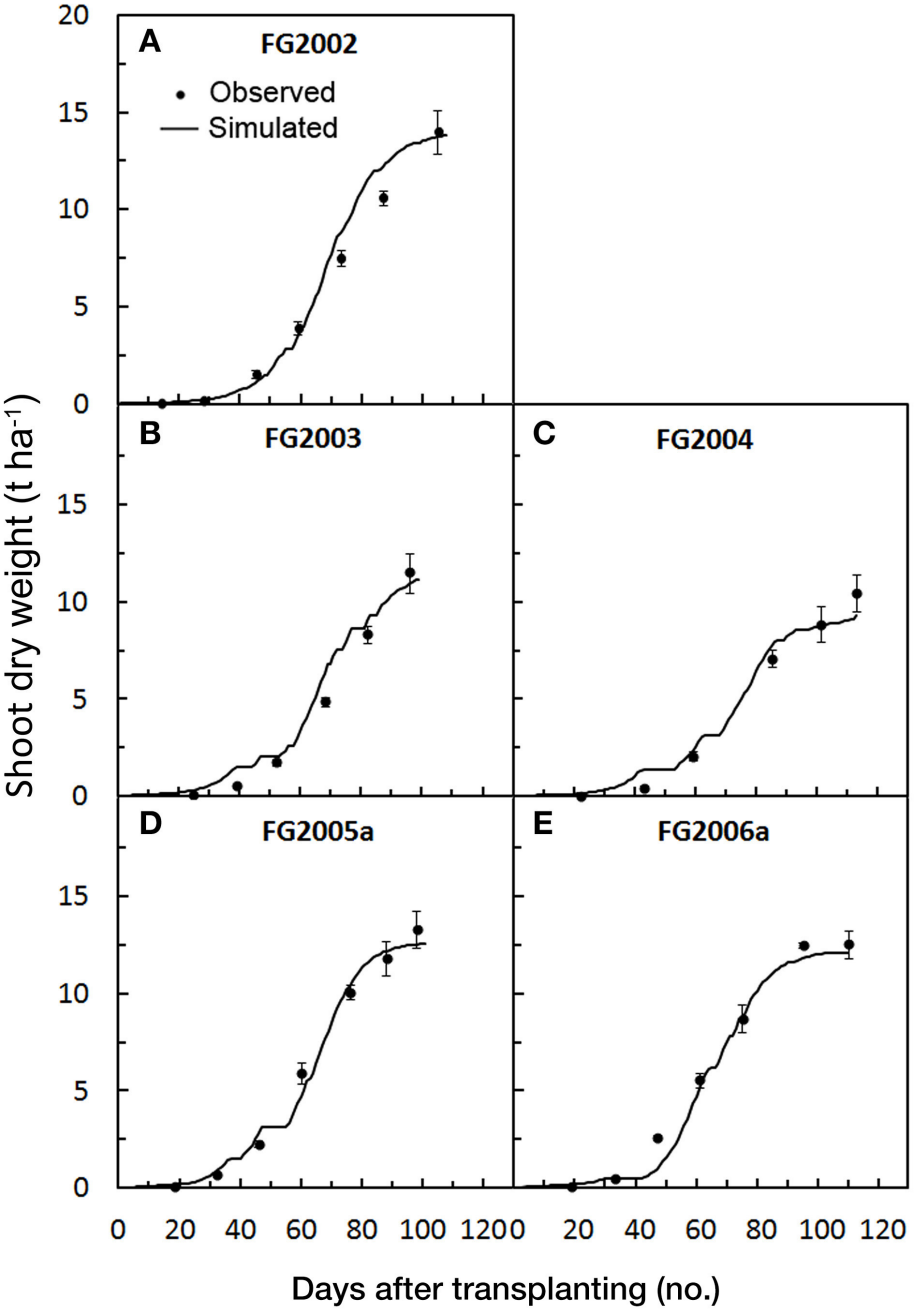
**Simulated and observed SDW accumulation during the growth cycle of the five tomato trials [FG2002 (A), FG2003 (B), FG2004 (C), FG2005a (D), and FG2006a (E)], used for calibrating** ***GesCoN***. Mean standard errors, when larger than the symbol, are represented by vertical bars.

Higher RRMSE, RSR and MAE were obtained in the period 2002–2004 compared with 2005–2006, with the highest values being recorded in 2003. The negative percent BIAS (PBIAS) indicates that in 2002–2004 the model slightly overestimated SDW accumulation, especially in 2003 (−11.5), while its positive values in 2005 (1.3), and particularly in 2006 (7.4) (Table 3), indicate a low model underestimation in these years, as also shown in Figures 1A–E.

The model overestimation and the higher magnitude of the errors observed in the first 3 years could be related with the N fertilization rate and management used in these trials, where 100 kg ha^−1^ of N were broadcasted in two applications (half before planting and half at the first fruit truss formation). This may have reduced SDW accumulation compared with the following 2 years when a higher N dose (200 kg ha^−1^) was applied by the more efficient fertigation system (Table 1). In the calibration trials, despite the year-on-year variability, the NSE was very close to the optimal value (1), further proving the goodness of the model fit.

**Figure 2:**
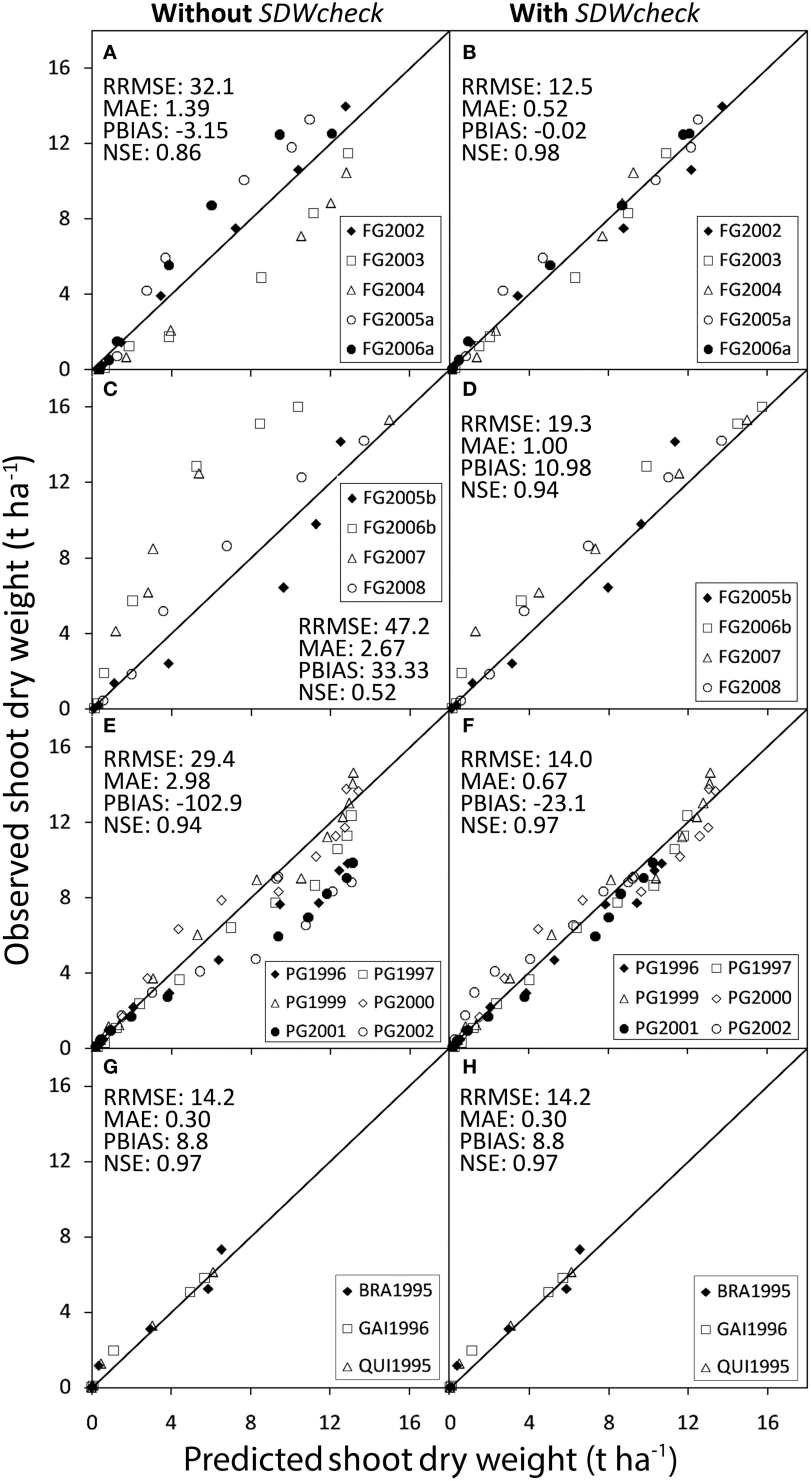
**Observed values of shoot dry weight against values predicted by** ***GesCoN*** **in the trials used for calibrating (A,B: Foggia calibration trials) and validating the software (C,D: Foggia validation trials; E,F: Perugia trials; G,H: Florida trials)**. For each location, represented by two boxes on the same row, the left box, refers to simulations performed without using the SDWcheck procedure and the right box, refers to the same simulations performed using the SDWcheck procedure.

The modeling performance was also strongly affected by the in-season *SDWcheck* calibration procedure, which largely compensates for the differences between cycles and greatly improved the goodness of fit (Figures 2A,B). On average, the *SDWcheck* calibration reduced the model error by 61% and the bias error by 99%, while improving the modeling efficiency by 14% (Figures 2A,B).

In terms of final values, a slight underestimation emerged in the simulation of the total SDW, the TFY and the cycle length, however, deviations were higher than 10% only in FG2004 for total SDW, in FG2005 for TFY and for cycle length FG2006. In almost all cases the deviations were very low, being below 5% (**Table 5**).

#### Nitrogen crop uptake

**Figure 3:**
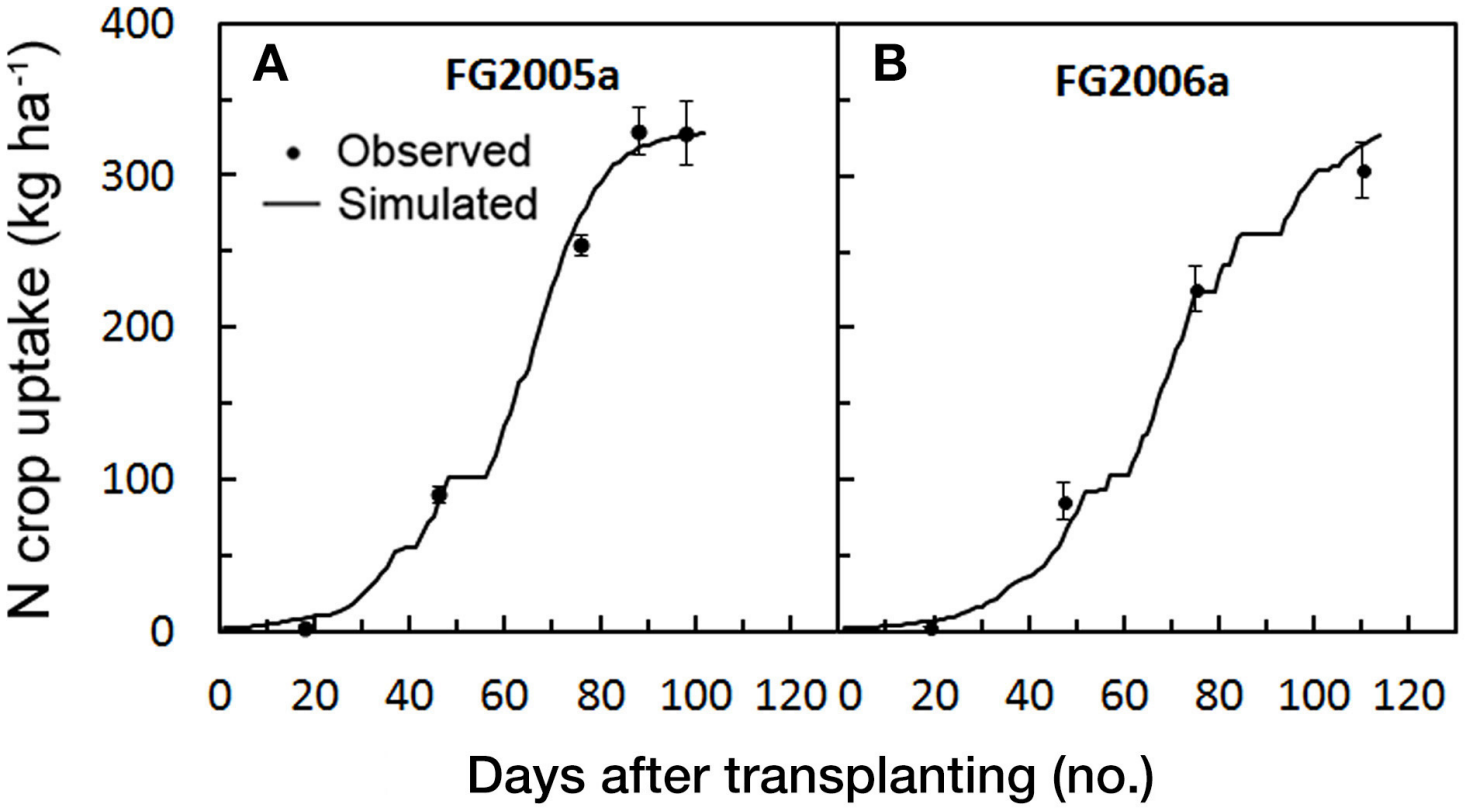
**Simulated and observed N crop uptake during the two tomato cycles in Foggia [FG2005a (A) and FG2006a (B)], used for calibrating** ***GesCoN***. Mean Standard errors, when larger than the symbol, are represented by vertical bars.

In the calibration trials where observed N uptakes were available (FG2005a and FG2006a), the magnitude of the model error was very low (Table 4). The averaged values of RMSE and MAE were 12.7 and 9.7 t ha^−1^, respectively. The RRMSE showed averaged values always below 10% (≈ 7%), and RSR was close to zero (0.03). There was not a systematic bias, and simulated N crop uptakes during the cycle roughly overlapped the observed ones with a very slight overestimation of the N uptake in 2005 (PBIAS = −1.78) (Figure 3). As a whole, all the indices together with the Nash-Sutcliffe efficiency close to 1, proved the model simulation to be excellent for the N crop uptake. Even in terms of final N uptake in both FG2005a and FG2006a the simulated values (311.1 kg ha^−1^, on average) were very close to the observed ones (316.4 kg ha^−1^, on average).

**Table 4:** **RMSE, Root mean square error; RRMSE, relative root mean square error; RSR, RMSE-observation standard deviation ratio; MAE, mean absolute error; PBIAS, percent bias; Nash-Sutcliffe efficiency (NSE) for the fittings performed on progression of N uptake in calibration and validation trials**.

**Trial**	**RMSE**	**RRMSE**	**RSR**	**MAE**	**PBIAS**	**NSE**
**CALIBRATION**						
FG2005a	12.22	6.07	0.030	8.61	−1.78	0.998
FG2006a	13.13	8.47	0.037	10.79	0.07	0.997
**VALIDATION**						
FG2007	27.52	11.78	0.071	28.83	7.44	0.986
FG2008	59.36	29.65	0.160	44.57	20.83	0.929
PG1997	13.23	2.54	0.06	11.12	1.26	0.965
PG1999	41.61	23.84	0.09	30.81	17.00	0.874
BRA1995	14.41	20.08	0.093	10.85	0.45	0.948
QUI1995	12.40	19.01	0.116	9.24	−7.10	0.946

### Validation

#### Shoot dry weight accumulation

The progression of the predicted against the observed SDW accumulation in the 13 crop cycles used for validating *GesCoN* are reported in **Figures 5–7** for the Foggia, Perugia, and Florida areas, respectively.

**Figure 4:**
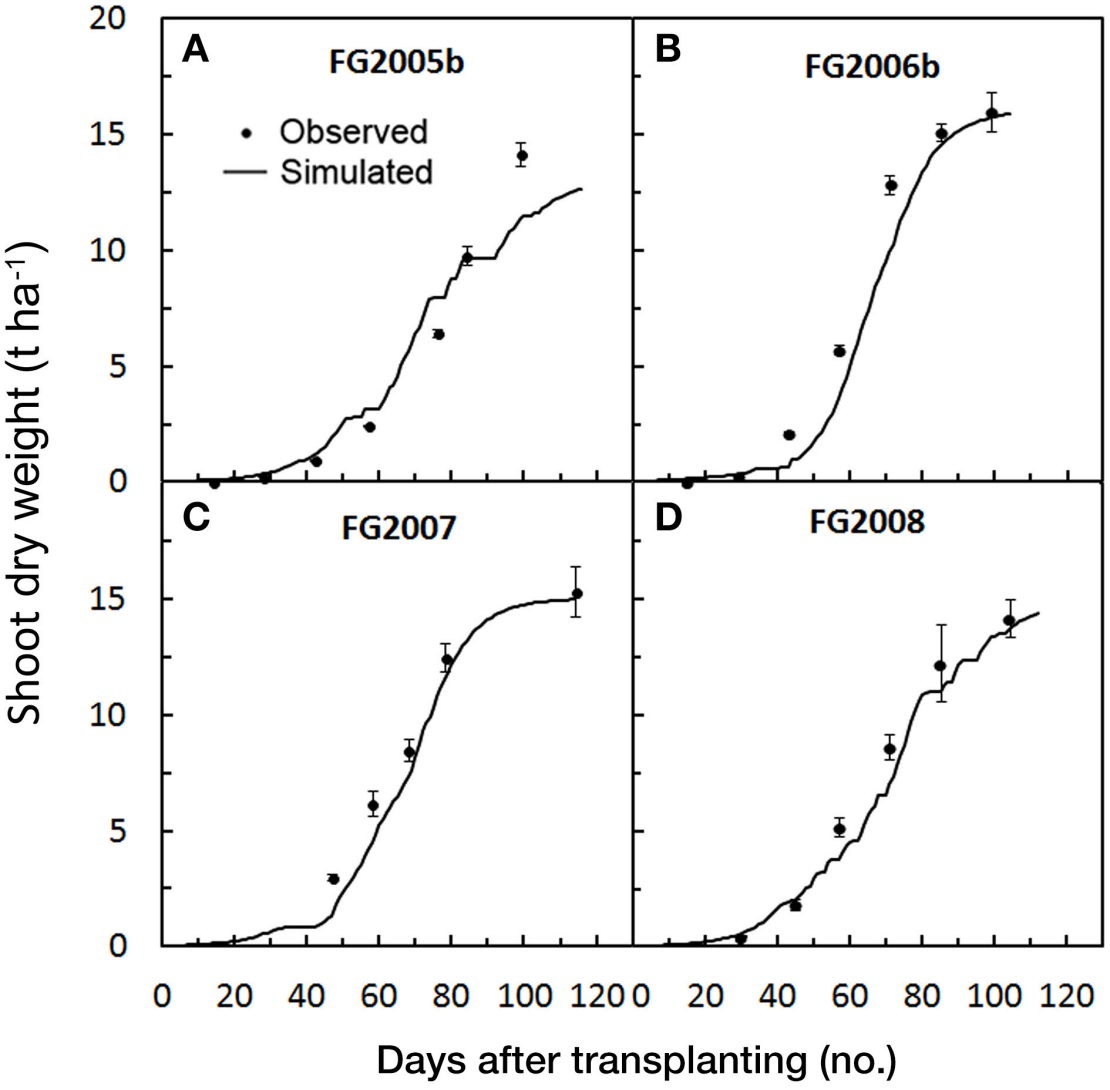
**Simulated and observed SDW accumulation during the four tomato cycles in Foggia [FG2005b (A), FG2006b (B), FG2007 (C), FG2008(D)], used for validating** ***GesCoN***. Mean standard errors, when larger than the symbol, are represented by vertical bars.

In the Foggia trials the averaged RMSE and MAE for SDW simulations were 1.2 and 1.0 t ha^−1^, respectively, while the RRMSE was 18.7%, on average. In the FG2005b trial, the RRMSE showed the highest value (26%) in contrast with the lowest MAE (Table 3). The large deviations which occurred in the last part of the FG2005b cycle (Figure 4A) may explain the high RRMSE value, which emphasizes the larger differences, compared with the low MAE value (0.81). The mean RSR was very close to 0 (0.1), while PBIAS values indicated a model underestimation of SDW particularly in the 2006–2008 trials, as can be inferred from Figures 4B–D. The calibration was performed on crops fertilized with N rates having optimal agronomical use efficiency (100–200 kg ha^−1^, not always through fertigation), which were lower compared with those used in the validation trials (250–300 kg ha^−1^, always through fertigation). It is likely that in the FG 2006–2008 trials a higher N soil availability resulted in a higher rate of crop biomass accumulation, also confirmed by the observed total SDW accumulation, which were generally slightly higher than the simulated ones (Table 5). However, in these simulations averaged NSE (0.95) still indicates an acceptable performance of the model in prediction of SDW.

**Table 5:** **Differences between the simulated and the observed total shoot dry weight (SDW), total N crop uptake, total harvested fruit yield (TFY), and cycle length of field grown tomato in the five trials carried out from 2002 to 2006 in the Foggia area, used for calibrating *GesCoN*., and in 13 trials from different years and locations (Foggia, Perugia, and Florida) used for validating *GesCoN***.

**Trial**	**Total SDW^a^**			**Total N uptake**			**TFY**			**Cycle length^a^**		
	**Obs^b^ (t ha^−1^)**	**Sim^c^ (t ha^−1^)**	**Dev^d^ (%)**	**Obs (kg ha^−1^)**	**Sim (kg ha^−1^)**	**Dev (%)**	**Obs (t ha^−1^)**	**Sim (t ha^−1^)**	**Dev (%)**	**Obs (DAT)**	**Sim (DAT)**	**Dev (%)**
**CALIBRATION**												
FG2002	14.0	13.8	−1.4	–	343.1	–	155.0	150.8	−2.7	113	108	−4.4
FG2003	11.5	11.1	−3.5	–	286.2	–	116.0	121.1	+4.4	109	99	−9.2
FG2004	10.5	9.1	−13.3	–	230.3	–	98.0	99.5	+1.5	113	112	−0.9
FG2005a	13.3	12.6	−5.3	328.6	316.6	−3.7	154.6	136.9	−11.4	111	101	−9.0
FG2006a	12.5	12.1	−3.2	304.3	306.6	+0.8	135.2	132.1	−2.3	118	104	−11.9
**VALIDATION**												
FG2005b	14.2	12.7	−10.6	–	262.7	–	116.5	138.0	+18.5	100	115	15.0
FG2006b	16.0	15.9	−0.6	–	385.0	–	127.6	173.3	+35.8	107	104	−2.8
FG2007	15.3	14.9	−2.6	351.1	365.1	+4.0	138.2	162.5	+17.6	109	106	−2.8
FG2008	14.2	14.4	+1.4	363.2	323.5	−10.9	153.3	157.0	+2.4	105	112	6.7
PG1996^f^	9.7	10.9	+12.4	–	224.8	–	120.8	130.4	+7.9	105	100	−4.8
PG1997^f^	12.4	12.1	−2.4	288.0	242.1	−15.9	163.1	145.3	−10.9	109	100	−8.3
PG1999^f^	14.5	13.1	−9.7	347.0	255.0	−26.5	181.1	157.3	−13.1	104	100	−3.8
PG2000^f^	13.5	13.4	−0.7	–	255.2	–	165.1^d^	161.1	−2.4	99^c^	102	+3.0
PG2001^f^	9.8	10.8	+10.2	–	217.0	–	134.3^d^	129.4	−3.6	91^c^	104	14.3
PG2002^f^	9.0	9.4	+4.4	–	204.1	–	–	112.3	–	102^c^	100	−2.0
BRA1995^g^	7.3	6.6	−9.6	147.9	160.0	+8.2	91.2^e^	78.9	−13.5	102	94	−7.8
GAI1996^g^	5.7	5.8	+1.8	–	147.7	–	60.5^e^	69.6	+15.0	91	101	+11.0
QUI1995^g^	6.1	6.4	+4.9	148.2	157.8	+6.5	65.1^e^	76.6	+17.7	84	99	+17.9

a *If not available from the original paper as yield data, the last sampled value with the relative simulated data is considered*.

b *Obs, observed value*.

c *Sim, simulated value*.

d Dev, deviation. It was calculated as: Dev = (predicted – observed)/observed * 100.

e *When harvesting date has not been reported by the authors, the value refers to the last sampling date, supposing that it was near to the harvest*.

f *Reported or calculated from Tei et al. (2002) and Onofri et al. (2009)*.

g *Reported or calculated from Scholberg et al. (2000a,b)*.

The in-season adjustment, through the *SDWcheck* procedure, performed after about one third of the cycle, greatly improved fits and gave a better estimate of SDW accumulation, thus providing the model with the flexibility to adapt to the different conditions. The step of growth rate adjustment was indeed intended as a strategy to sum up and to cope with the specific pedoclimatic and crop conditions, including the diverse genetic features. In the Foggia validation trials, the *SDWcheck* in-season calibration procedure (Figures 2C,D) was effective in reducing the model error by more than 60%, the bias error by more than 66% and in improving the NSE model efficiency index by about 78%.

**Figure 5:**
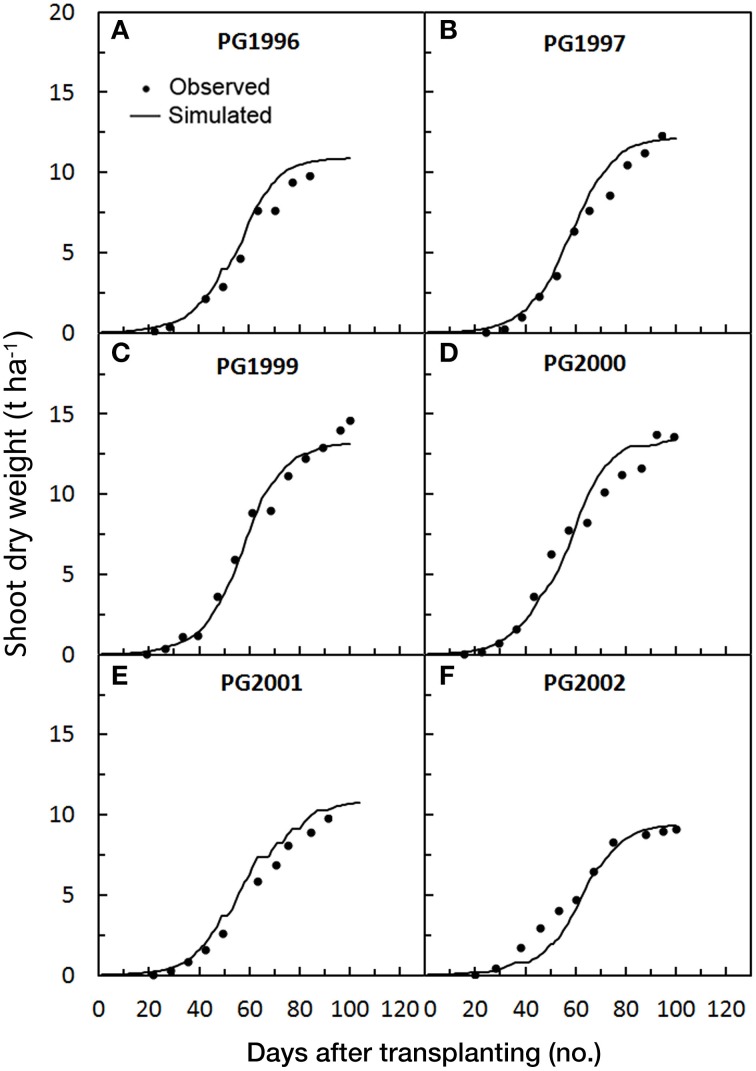
**Simulated and observed SDW accumulation during the six tomato cycles in Perugia [PG1996 (A), PG1997 (B), PG1999 (C), PG2000 (D), PG2001 (E), PG2002 (F)], used for validating** ***GesCoN***. Mean standard errors, when larger than the symbol, are represented by vertical bars. (Re-elaborated from Tei et al., 2002; Onofri et al., 2009).

In the Perugia trials, the magnitude of model error in the simulation of SDW accumulation was, on average, lower than the Foggia ones (RMSE = 0.81 t ha^−1^; MAE = 0.61 t ha^−1^; RRMSE = 14.2%), with a RSR closer to zero (0.06). The model gave a general slight overestimation (PBIAS = −3.6%, on average), probably due to the application in these trials of all the N fertilizer before transplanting, which could have reduced the N efficiency compared with calibration trials. However, in PG2002 an underestimation was only evident in the first part of the cycle and in PG1999 an underestimation was detected in the final part of the cycle, probably due to the high rate used in this trial (400 kg ha^−1^) (Table 1). The total SDW deviations were acceptable and ranged from –9.7 to +12.4% (Table 5). The model performed well in all the Perugia simulations, with NSE = 0.97 (Table 3, Figure 5).

Even in this group of data the *SDWcheck* procedure improved the model fit. The in-season calibration was able to reduce both the bias error (by 77.5%) and the model error (the RRMSE by 52.5% and the MAE by 77.5%), with a consequent improvement in modeling efficiency (+3.2%) (Figures 2E,F).

**Figure 6:**
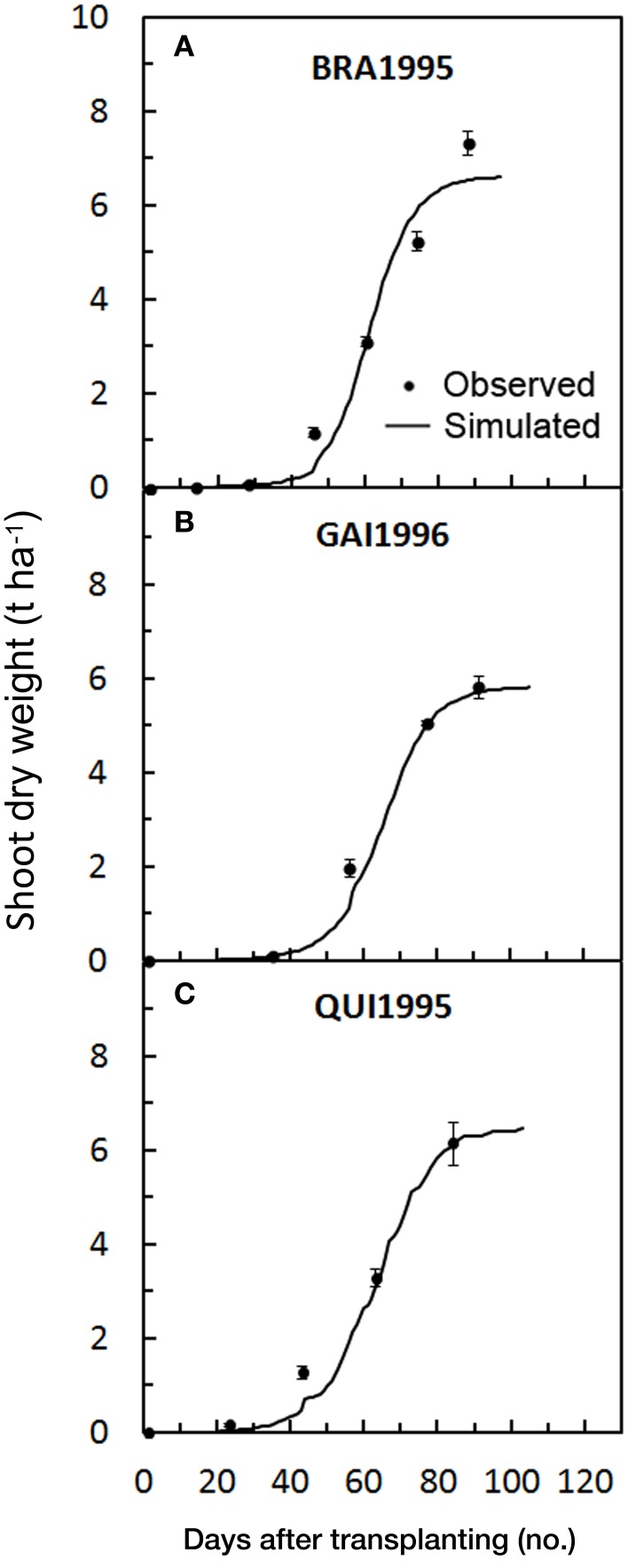
**Simulated and observed SDW accumulation during the three tomato cycles in Florida [BRA1995 (A), GAI1996 (B), QUI1995 (C)], used for validating** ***GesCoN***. Mean standard errors, when larger than the symbol, are represented by vertical bars. (Re-elaborated from Scholberg et al., 2000a,b).

The SDW accumulation for Florida trials was satisfactorily predicted by the model, showing averaged RMSE and MAE of 0.44 and 0.30t ha^−1^, respectively. The values of RRMSE were lower than the threshold of 20% (17.3% on average) and the mean RSR was 0.079. The model showed a slight underestimation (PBIAS = 9.1%, on average), particularly in the final part of the BRA1995 cycle (Figure 6A), resulting in lower total SDW prediction (Table 5). The general underestimation could be explained by the larger fruit size of the cultivars used in Florida trials, compared with those used in the calibration trials. In BRA1995 the irrigation system used (seepage) might also have emphasized the yield potentiality of the cultivar in this trial. Despite these small deviations, modeling efficiency was very high (NSE = 0.97). The *SDWcheck* in-season calibration did not produce any improvement in any of the Florida trials (Figures 2G,H). In these trials, instead, the correct assessment of the stage of seedlings at transplanting played a key-role in improving the SDW simulation. According to the available data, a dry weight of plantlets at transplanting of 0.2 g was assessed, hence determining a longer lag-phase at the beginning of the simulation and thus a better synchronization of Florida cycles with those used in the calibration.

Despite the high level of simplification used in modeling the accumulation of SDW, which is only based on the thermal sum (Elia and Conversa, 2015), there was a general good agreement between simulated and observed SDW accumulation in the different validation trials, which were collected under different environmental conditions and with different management practices and genotypes.

#### Nitrogen crop uptake

**Figure 7:**
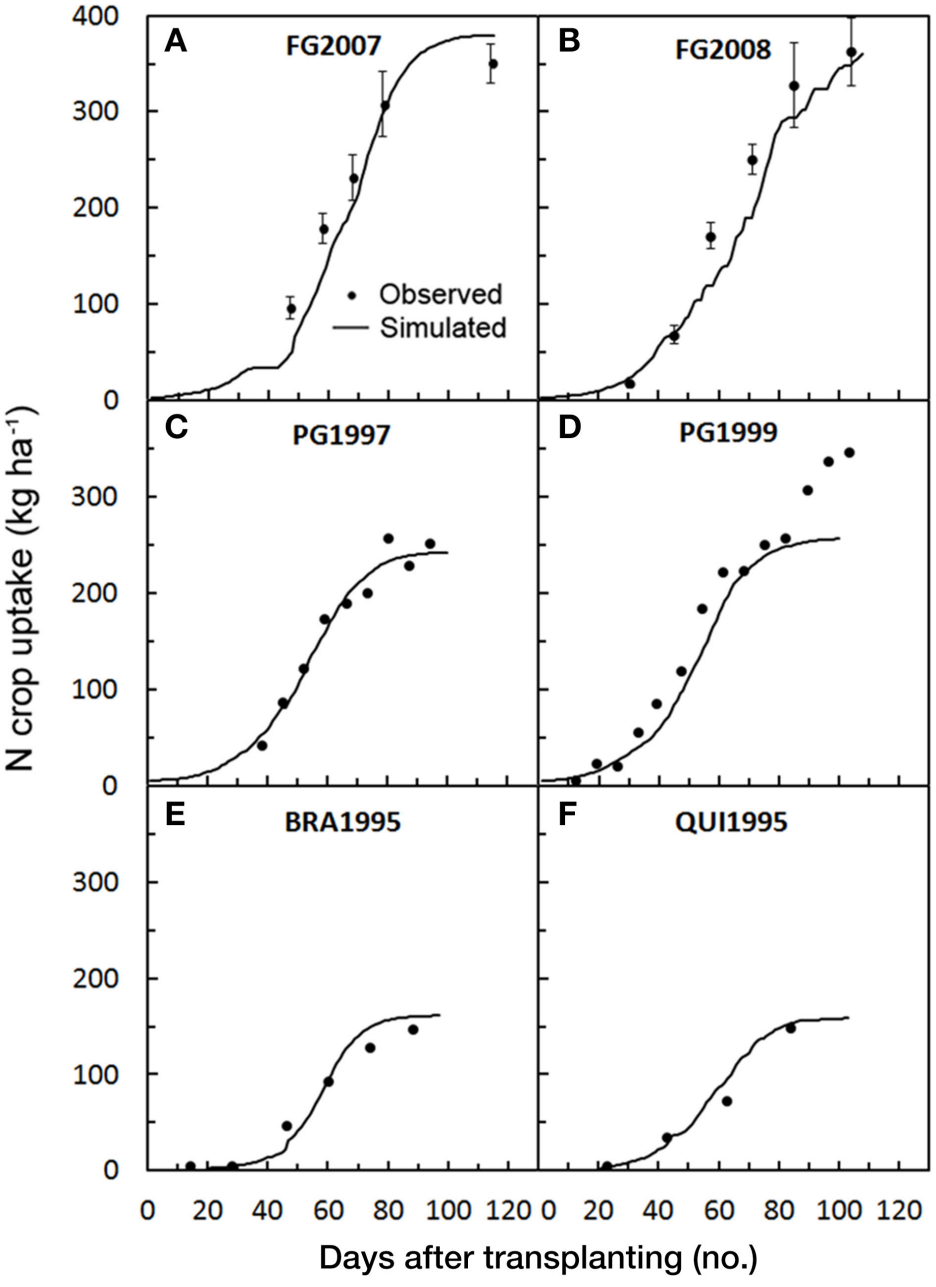
**Simulated and observed N crop uptake during the tomato cycles in Foggia (A: FG2007; B: FG2008), in Perugia (C: PG1997; D: PG1999) and in Florida (E: BRA1995; F: QUI1995), used for validating** ***GesCoN***. When available, mean standard errors are represented by vertical bars. (PG1997 and PG1999 re-elaborated from Tei et al., 2002; Onofri et al., 2009; BRA1995 and QUI1995 re-elaborated from Scholberg et al., 2000a,b).

Among all the validation trials, in FG2008 the model prediction was above the observed N uptake and showed the highest value of RMSE, RRMSE, RSR, MAE, and PBIAS and a very low NSE (Table 4). In FG2008 starting from 40 days after transplanting onward, the model moderately underestimated N crop uptake, especially in the period of rapid growth (PBIAS = 20.8%, Table 4) (Figure 7B), while in FG2007 the underestimation (Figure 7A) was less evident (PBIAS = 7.4%). Both these trials were fertigated with higher N rate (300 and 400 kg ha^−1^ in 2007 and 2008, respectively) as compared with the calibration ones, justifying the general underestimation of the model. This is also confirmed by the fact that the observed total crop N uptakes were 351 in FG2007 and 363 kg ha^−1^ FG2008, and simulated total N uptake in FG2008 were 10.9% lower than the observed ones (Table 5). Despite the large model errors in 2008, the averaged NSE = 0.96 (Table 4) indicates a good modeling efficiency.

In PG1997, the N crop uptake simulation showed a very low magnitude of model error (RRMSE = 2.5%; RMSE = 13.2 t ha^−1^; MAE = 11.1 t ha^−1^) and a very low underestimation (PBIAS = 1.3%), with a high modeling efficiency (NSE = 0.97). In the PG1999 simulation the model only failed in the prediction of N uptake in the last quarter of the crop cycle with a large underestimation (Figures 7C,D), resulting in moderately high PBIAS (17.0%) and model error (RMSE = 41.6; RRMSE = 23.8; MAE = 30.8). As a consequence the modeling efficiency was reduced, resulting the lowest value among all the validation trials (NSE = 0.87) (Table 4). The scarce model fitting performance in PG1999 which occurred in the last part of the cycle can be related to the high N fertilization rate used in this trial(400 kg ha^−1^ of N in the form of ammonium nitrate) (Table 1), as above reported to explain the model underestimation of SDW. Unexpectedly, in the last part of the cycle, when N uptake in a processing tomato approaching maturity is normally declining, the authors reported an increasing N uptake trend. It is probable that both the high N rate used and a possible delay in the nitrification process had allowed a large nitrate availability in the soil late in the season, which might have boosted plant growth and plant N uptake in the last part of the cycle. In PG1999 N the total crop uptake was 347 kg ha^−1^, underestimated by 26.5% by the model (Table 5).

N plant uptake was well-predicted in all Florida simulations. Model error was on average relatively low (RSME = 13.4, RRSME = 19.5, MAE = 10.0, RSR = 0.10, on average) with a good model efficiency (NSE = 0.95) and a slight overestimation (PBIAS = −3.32%). Observed total N uptake (148.0 kg ha^−1^ on average) was also overestimated.

#### Fruit yield and cycle duration

In the Foggia trials, fresh harvested fruit yield (TFY) showed simulated values higher than observed ones, particularly in FG2006b where the overestimation was 35.8%. The positive deviations in TFY prediction could be linked to a slight model underestimation of the effects of stress weather conditions on reproductive traits. In Foggia hot weather conditions during flowering and fruit-set stages were, indeed, more frequent in validation than in calibration trials (personal communication).

The model simulations in the Perugia area more frequently showed an underestimation of the TFY (–7.5%), which was higher (–13.1%), as expected, in the 1999 trial. In the BRA1995 trial there was an underestimation of both total SDW and TFY which could be linked, as reported above for SDW accumulation, to the combination of the irrigation system used (seepage) with a large fruit-sized cultivar. For GAI1996 and QUI1995 trials, instead, total SDW was very well-predicted (+3.3% deviation, on average), while TFY was more largely overestimated (+16%, on average). This greater deviation in TFY may be linked to the unique harvest index (HI) (fruit dry weight/total above-ground dry weight at harvest) used in all the simulations (0.66). The authors have, indeed, reported for Gainesville and Quincy HI values of 0.56 and 0.50, respectively (Scholberg et al., 2000a). In general, the TFY prediction was quite good, although less efficiently simulated than total SDW, underlining that a better assessment of HI for the specific cultivar could allow a better estimation of TFY. Considering all the trials, the estimation of the time to harvest (cycle length) may be evaluated as generally good and in some cases excellent. The deviations were only higher than 10% in four cases out of thirteen, in three cases they were between 5% and 10% and in six cases lower than 5% (Table 5).

#### Soil water content

**Figure 8:**
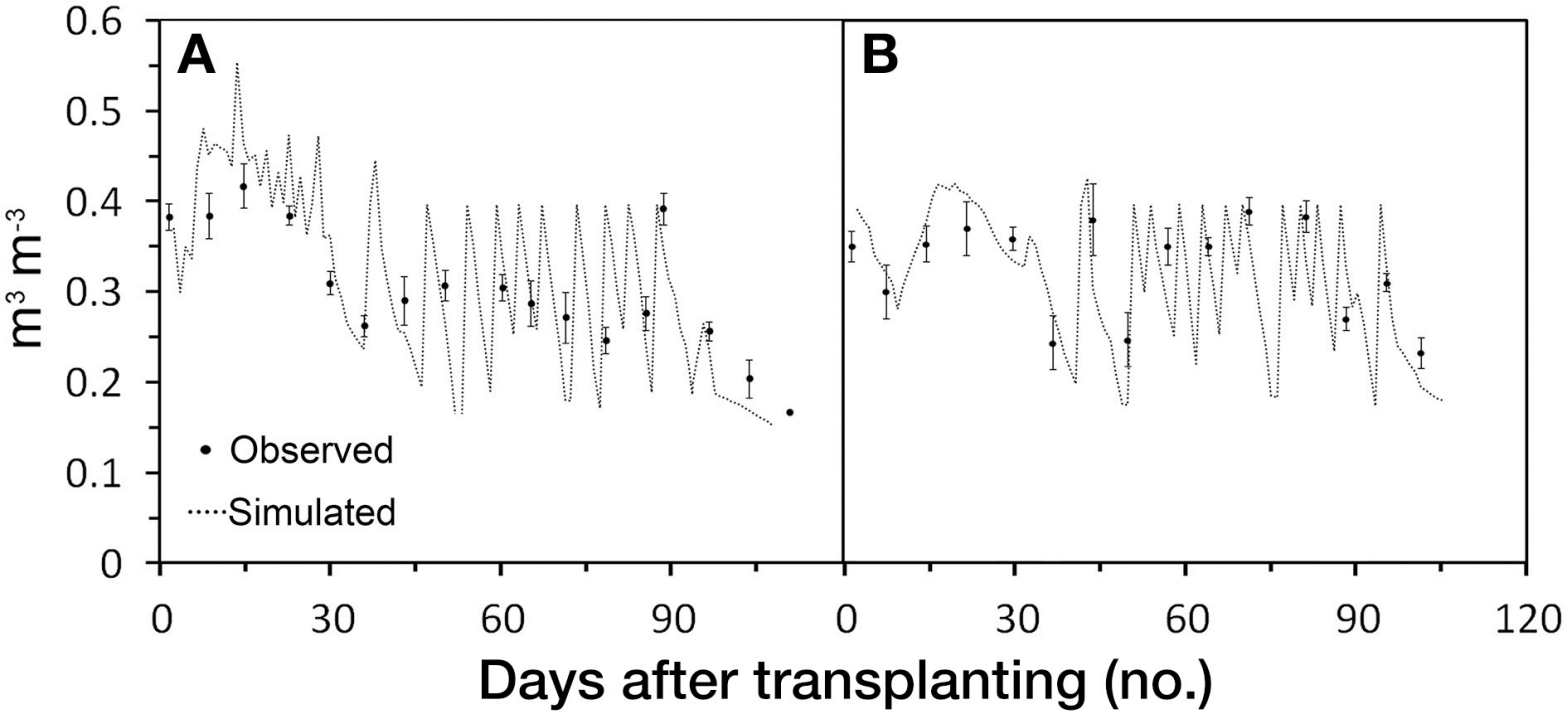
**Simulated and observed volumetric soil water content during growing season in FG2005b (A) and FG2006b (B) trials**. Standard deviations, when larger than the symbol, are represented by vertical bars.

Under the boundary conditions used by *GesCoN*, the soil water content (SWC) appears to be well-simulated by the software. At the 10–30 cm depth, the most relevant layer for plant water uptake, SWC appears to be well-described by the DSS, also considering the quite large standard deviation ranges (Figure 8). However, considering that SWC was only tested on a limited set of data (2 trials) obtained on the same type of soil (loamy), further investigations should test the software under different pedoclimatic conditions in order to confirm its performance.

### *GesCoN* response under different N soil availability scenarios

**Figure 9:**
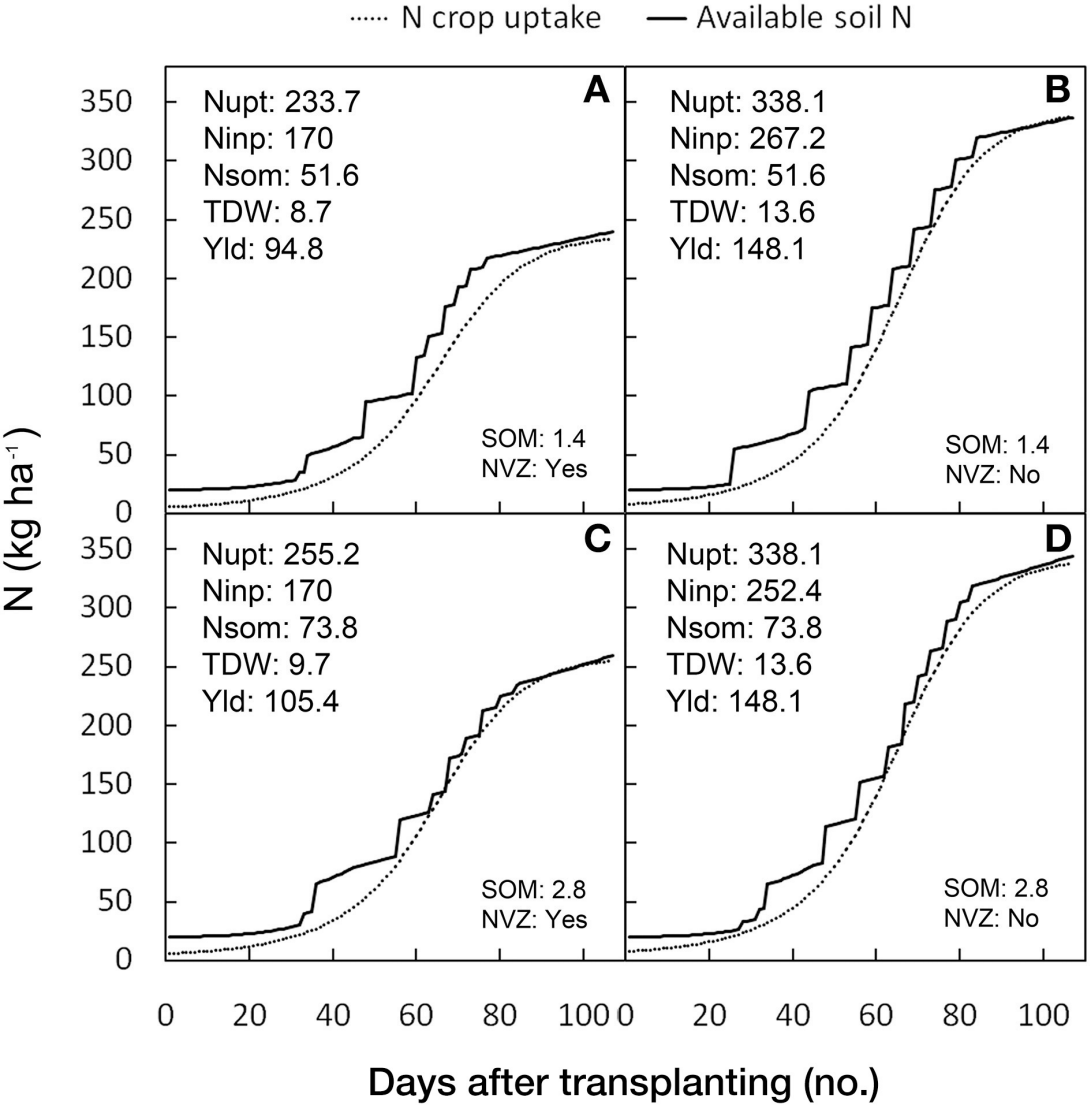
**Prevision by** ***GesCoN*** **of N crop uptakes, N application schedules, and N soil availability under four different N scenarios**. The simulations were performed with the same soil, climatic and cropping conditions and changing the level of the soil organic matter (SOM) [SOM: 1.4 g 100 g^−1^, top graphs: **(A,B)**; SOM: 2.8 g 100 g^−1^, bottom graphs: **(C,D)**] and the NVZ condition (NVZ: “No,” left graphs; NVZ: “Yes,” right graphs). In each box the simulated total crop N uptake (Nupt, in kg ha^−1^), total N input (Ninp, in kg ha^−1^), total N from SOM mineralization (Nsom, in kg ha^−1^), total aboveground DW (TDW, in t ha^−1^), and total fresh yield (Yld, in t ha^−1^), are also reported.

The results of the four N scenarios are represented graphically in Figure 9. The N scenarios had the same soil, climatic and growing conditions, with differences in the level of the SOM (1.4 or 2.8 g 100 g^−1^ of soil) and whether or not they were in an NVZ area. It can be seen that when the DSS works without N fertilizer limitation (non NVZ area) (Figures 9B,D), the maximum TDW (13.6 t ha^−1^) and yield (148.1 t ha^−1^) and N uptake (338.1 kg ha^−1^) can always be achieved irrespective of the SOM level. However by increasing the SOM from 1.4 to 2.8 g 100 g^−1^ of soil, the need for N fertilizer input is reduced by 5.6%. When the simulations were performed in NVZ areas (where maximum N supply is limited to 170 kg ha^−1^) the DSS simulates a reduction in N uptake by 30.9 and 24.5% in the lower compared with the higher SOM condition, corresponding to a yield reduction of 35.9 and 28.8%, respectively (Figures 9A,C).

These simulations prove the capacity of the software to adapt to different N availability scenarios by modifying the N fertilization schedule through the simulation of the crop response at limiting N availabilities and the adaptive control of the maximum potential yield.

## Conclusions

The DSS *GesCoN* has been calibrated on a high yielding processing tomato hybrid fertilized with N rates and N distribution modalities assuring high N use efficiency. The DSS performed very well, particularly when validation trials had similar N fertilization conditions and cultivar typology of the calibration ones. Underestimations both in SDW and N uptakes were found when high N rates were used (FG2007, FG2008, and PG1999) or when the seepage irrigation, typically affecting crop growth because of the improved nutrient availability, was used. Overestimations were found when the modalities and/or the rates of N application reduced the N soil availability during the crop cycle, such as when all N was broadcasted in a single pre-planting application (e.g., PG trials).

In general the DSS performed in a more than acceptable way, even if growth modeling is based on an empirical regression model with only the thermal sum as an independent variable. It proved to be good in simulating SDW accumulation and N uptake of tomato crops conducted with different genotypes and over a quite large number of years both under Mediterranean and subtropical conditions. The in-season “*SDWcheck*” procedure greatly contributed to improving its growth prediction under the different pedoclimatic and genetic conditions.

The DSS proved to control the potential DW accumulation under different N soil availability scenarios and to adaptively modulate N fertilizer application in order to optimize the crop performance, even under limiting levels of N availability.

In terms of fruit yield, deviations between observed and predicted values were recorded when the cultivar typology was quite different compared with the calibrated genotype (e.g., the fresh tomato hybrids used in Florida characterized by large sized fruits). In these cases, a better assessment of harvest index could significantly improve fresh yield prediction. Under the boundary conditions used by *GesCoN*, the soil water content (SWC) appears to be well-simulated by the software.

The calibrated parameters may need to be further adjusted as the model is further tested against additional data sets, and also as the model structure or algorithm is improved in future versions.

Further investigations with field experiments designed to produce appropriate input and output data must be undertaken to validate the performance of *GesCoN* in the prediction of the soil N and the humidity level.

### Conflict of interest statement

The authors declare that the research was conducted in the absence of any commercial or financial relationships that could be construed as a potential conflict of interest.
